# ATTITUDES, EXPECTATIONS, KNOWLEDGE AND INTENTIONS REGARDING ORAL HEALTH: PERCEPTIONS OF OLDER ADULTS

**DOI:** 10.1093/geroni/igz038.3188

**Published:** 2019-11-08

**Authors:** Virginia M Hardgraves, Jean Henry, Susan K Patton

**Affiliations:** University of Arkansas Fayetteville, Fayetteville, Arkansas, United States

## Abstract

Many adults in today’s aging cohort are maintaining their teeth into their advanced years. The advantages of fluoridated water, dental insurance, greater awareness of preventive oral healthcare, and more restorative dental services, have made this possible. The demand for oral health care services will be greater and more complex than that of previous generations. Evidence of a link between oral health and overall health underscores the need to better integrate dental care into the healthcare system. The aim of this study was to better understand these issues from the perspective of older adults (N = 26) 65 years of age and older and living independently. Semi structured interviews guided by the behavioral constructs of the Reasoned Action Approach Theory were conducted. Results from the qualitative analysis revealed five themes: 1) Difficulties accessing dental care, 2) Stoic independence, 3) Taking care of your mouth as part of overall health, 4) Relationships affecting oral health related quality of life, and 5) Supporting roles. The findings demonstrate a need to increase oral health literacy in the older adult population with attention to reducing modifiable risk factors. Understanding these behaviors and the current level of oral and overall health knowledge from the perspective of older adults, is vital to helping these individuals’ transition into increasing levels of dependency with a high level of oral health related quality of life. Public health program planning can use this information to help older adults prepare for the transitions that come with healthy aging.

